# The bumpy road to achieve reliability of clinical profile characteristics in psychosis and related disorders

**DOI:** 10.1002/mpr.1858

**Published:** 2020-12-08

**Authors:** Steven Berendsen, Mirjam J. van Tricht, Amy Tedja, Thijs J. Burger, Mariken B. de Koning, Lieuwe de Haan

**Affiliations:** ^1^ Department of Psychiatry University Medical Center Amsterdam Location Academic Medical Center Amsterdam The Netherlands; ^2^ Arkin Mental Health Care Amsterdam The Netherlands

**Keywords:** clinical profiling, heterogeneity, inter‐rater reliability, psychosis, training procedures

## Abstract

**Objectives:**

Profile characteristics are factors that are relevant for diagnosis, prognosis or treatment. The present study aims to develop a set of clinically relevant profile characteristics. Moreover, our goal is to determine the inter‐rater reliability (IRR) of the selected profile characteristics.

**Methods:**

Potential profile characteristics were determined by literature review. Assessment of IRR was done by comparing scores on profile characteristics determined by two researchers. We conducted three subsequent studies: (1) assessment of pre‐training IRR, (2) IRR following implementation of an instruction manual, (3) IRR after optimizing scoring methods. IRR was measured with the Intraclass Correlation Coefficient (ICC).

**Results:**

IRR scores of profile characteristic Illegal activities were high across the three studies (ICC ≥ 0.75). Following training procedures in study 2 and 3, reliability estimates remained low to moderate (ICC < 0.75) for the profile characteristics Support of relatives, Aggression recent and lifetime, substance use and insight recent. IRR scores of the other eight profile characteristics varied from low, moderate to high across studies.

**Conclusion:**

IRR scores of profile characteristics were highly variable, and mostly inadequate in all three studies. Consequently, further research should focus on specification of severity scores of profile characteristics, optimizing scoring methods and re‐evaluation of IRR.

## 1 INTRODUCTION

The heterogeneity of schizophrenia and related disorders is omnipresent. Manifestations of the disease vary strongly in terms of symptomatology, vocational functioning and social participation (Kahn et al., 2015; Owen, Sawa, & Mortensen, 2016). Furthermore, incidence and prevalence of psychiatric and somatic co‐morbidity vary between patients, contributing to the heterogeneity of diagnosis, prognosis and treatment (Sprah, Dernovsek, Wahlbeck, & Haaramo, 2017; Yum, Hwang, Nasrallah, & Opler, 2016).

To capture this substantial variation, new methods are being developed to provide a more precise diagnosis, examples are network analysis (Borsboom & Cramer, 2013), the trans‐diagnostic approach (Nelson, McGorry, Wichers, Wigman, & Hartmann, 2017) or clinical profiling (Wigman et al., 2013). These novel methods aim to disentangle the diverse presentation and outcome parameters of the disease into specific categories, which are relatively easy to recognize and may form a target for intervention (Kahn & Keefe, 2013). Eventually, the ultimate goal is to develop diagnostic tools that can improve prediction of outcome, individualized treatment and prevention of illness progression.

Clinical profiling have been proposed to create a comprehensive set of clinical relevant prognostic factors (Wigman et al., 2013). Profile characteristics may represent current as well as life time patient characteristics, relevant for diagnosis and prognosis. By profiling we may personalize the diagnosis by targeting the large variation in symptomatology, comorbidity and social participation.

Clinical profiling of psychiatric illness may offer various benefits at the level of scientific research and clinical practice, in comparison to other methods that aim to address heterogeneity. First, a set of clinically relevant factors could support clinicians to ensure quick recognition of specific problem areas and specify their treatment‐plan according to these profile characteristics. In fact, clinical profiling surpasses the current diagnostic categories and can be utilized in the trans‐diagnostic approach. Second, profile characteristics can be easily applied by clinicians at individual patient‐level, in comparison to the network approach which is predominantly applied on group‐level symptoms in research (Bos & Wanders, 2016). To perform network analysis on individual patient level longitudinal time series of experienced sampling are needed (Yang et al., 2018). Third, profiling in scientific research could provide a more comprehensive and detailed description of patient characteristics, and thereby contribute to the selection and comparison of study populations. Last, for several of the proposed profile characteristics specific therapeutic interventions are available, such as interventions to enhance social interactions, health education or medication adherence training (Abdel Aziz et al., 2016; Dodell‐Feder, Tully, & Hooker, 2015).

Until now a comprehensive overview of profile characteristics for psychosis and related disorders is missing. In addition, no studies have yet addressed the inter‐rater reliability (IRR) of assessing profile characteristics. Assessment of IRR is an important first step in the psychometric evaluation, considering that sufficient reliability of profile characteristics is a pre‐requisite for any further validity research (Kobak, Kane, Thase, & Nierenberg, 2007). Additionally, poor reliability leads to high variability of data and reduced power to find relevant differences (Perkins, Wyatt, & Bartko, 2000). An important tool to achieve sufficient reliability is training of raters, and this training should include information about profile characteristics, specifications for ratings and feedback on performance by experienced clinicians. Typically, training of raters leads to an increase in reliability estimates (Kobak, Engelhardt, Williams, & Lipsitz, 2004).

Consequently, in the present study we first aim to develop an overview of clinically relevant and feasible profile characteristics, and subsequently determine the IRR of these profile characteristics in patients presenting with recent onset psychotic symptoms. Third, we set out to evaluate whether training procedures for raters improve IRR. We hypothesize that IRR scores of profile characteristics without training procedures will be insufficient, and training of raters will substantially improve reliability estimates.

## 2 METHOD

### 2.1 Sample characteristics

In Table 1 the demographical and clinical characteristics of the separate studies are shown. In total we included 139 patients, of whom we included 99 patients in study 1, 20 patients in study 2 and another 20 patients in study 3. We included a smaller sample in study 2 and 3 as we only aimed to evaluate the effect of training procedures on IRR. The majority of patients were diagnosed with a schizophrenia spectrum disorder and used antipsychotic medication. No significant differences in age, gender, global assessment of functioning (GAF), use of antipsychotic medication or diagnosis were found between the samples that participated in the three studies.

**TABLE 1:** Demographic and clinical characteristics

	Study 1 (*n* = 99)	Study 2 (*n* = 20)	Study 3 (*n* = 20)	Between‐groups	*p*‐value
Gender (% male)	68.04%	55.00%	80.00%	*X* ^2^ (2.502)	0.286
Age (SD)	24.32 (6.0)	25.47 (5.14)	23.50 (3.92)	*F* = 0.056	0.946
GAF mean (SD)	52.28 (15.48)	50.65 (16.65)	50.00 (14.53)	*F* = 0.147	0.864
% Use of antipsychotic medication	86.67%	85.00%	73.33%	*X* ^2^ (0.724)	0.696
Diagnosis	‐	‐	‐	*X* ^2^ (4.697)	0.320
% Schizophrenia spectrum disorders	84.69%	85.00%	73.33%	‐	‐
% Bipolar disorder	9.18%	10.00%	26.67%	‐	‐
% Other psychotic disorders	6.12%	5.00%	0.00%	‐	‐

Abbreviations: GAF, global assessment of functioning; SD, standard deviation.

### 2.2 Design

The design of the current study was cross‐sectional and it contained three sub‐studies. We included outpatients from the diagnostic center of the Early Psychosis Unit at the department of psychiatry, at the University Medical Center Amsterdam, location Academic Medical Center. Three independent clinicians (authors AT and TJB, IL in the acknowledgments) performed inter‐rater reliability assessments across the sub‐studies.

### 2.3 Procedure

The main reason for referral to our diagnostic center was recent onset of psychotic symptoms. The diagnostic interview was conducted by a psychiatry resident or psychologist in training for healthcare psychologist, supervised by a clinical psychologist and psychiatrist. The psychiatrist and clinical psychologist were present during the first 30 min of the interview. After the diagnostic interview a comprehensive formal report was written by the psychiatry resident or psychologist in training, supervised by the psychiatrist or clinical psychologist. This report was written in a predefined format, and included the current psychiatric condition, illness history, information from relatives and other relevant information for diagnosis and treatment. It was a comprehensive document of approximately four to six pages. This formal report based on the diagnostic interview was used for IRR purposes.

### 2.4 Profile characteristics

We selected clinically relevant profile characteristics based on an extensive review performed by the committee that developed the clinical guidelines for the diagnosis and treatment of psychotic disorders in the Netherlands (Trimbos, 2019). To substantiate the latter review we evaluated whether the selected profile characteristics in psychosis and related disorders were significantly associated with increase in psychotic symptoms or psychiatric re‐hospitalization in follow‐up studies (Alvarez‐Jimenez et al., 2012; Braehler et al., 2013; de Haan, van Amelsvoort, Dingemans, & Linszen, 2007; Emsley, Chiliza, Asmal, & Harvey, 2013; Kooyman, Dean, Harvey, & Walsh, 2007; Michalska da Rocha, Rhodes, Vasilopoulou, & Hutton, 2018; Peter, Weiden, Grogg, & Locklear, 2004; Rabinovitch, Bechard‐Evans, Schmitz, Joober, & Malla, 2009).

Thereafter, our research group “Severe Psychotic Disorders of the University Medical Center Amsterdam of the University of Amsterdam and Arkin,” including psychiatrists and psychologists with extensive clinical and scientific experience with psychotic disorders, provided input on clinical relevance of profile characteristics and on the feasibility to assess profile characteristics in regular practice. The selection of profile characteristics was founded on three aspects: (1) profile characteristics needed to be related to an increase in psychotic symptoms or psychiatric re‐hospitalization, (2) profile characteristics should be easily applicable by clinicians and (3) profile characteristics should be clinically relevant for outcome measures in schizophrenia spectrum disorders. This resulted in the final set of profile characteristics that were used in our study.

### 2.5 Inter‐rater reliability

The general procedure of IRR assessment was as follows: two clinicians independently determined severity scores of profile characteristics by thoroughly evaluating the formal report written after the diagnostic assessment. The clinicians did not see the patient and only had access to the formal report. IRR was assessed by comparing severity scores of profile characteristics determined by both clinicians. We chose this procedure because it is in line with clinical practice in which clinicians frequently base decisions on written information of other colleagues. Moreover it is in accordance with previous studies concerning reliability of medical diagnosis based on electronic record review (Chung, Chiang, Chou, Chu, & Chang, 2010; Kang et al., 2013; Varmdal et al., 2015). Estimation of IRR by multiple raters who merely observe video fragments may reduce information variance, leading to artificially inflated reliability estimates (Kobak et al., 2004). To evaluate the influence of training procedures on the IRR of profile characteristics, we conducted three subsequent studies:

Study 1 (*n* = 99)

Pre‐training IRR was determined by comparing severity scores of profile characteristics assessed by two independent researchers (A.T and I.L., mentioned in the acknowledgments). Both researchers had access to the description of the profile characteristics as shown in Table 2, there was no other training or instruction beforehand.

**TABLE 2:** Description of profile characteristics

Profile characteristic	No/very mild problems	Moderate problems	Serious problems
Work or other activities	A paid job or equivalent of daily activities, e.g., volunteering, study, care taker tasks	Some structural activities, for instance work, volunteering, study/school, sports, social activities or care taker tasks for 2 days per week	Less than 2 days per week with structural activities
Support system	Adequate support by family/friends/partner, no or minimal problems	Moderate support or moderate problems, for instance dedicated support available only by 1 person or when support leads to infrequent conflicts	No or minimal support or serious problems or conflicts
Physical health	No or minimal physical health issues	Moderate physical health problems for instance being at risk for physical health problems, for instance metabolic complications of antipsychotic medicine or physical illness with moderate impact on functioning, for instance joint problems or well‐regulated diabetes	Serious physical health problems with impact on daily life, for instance diabetes that is difficult to regulate
Substance usage (excluding nicotine) Lifetime Last month	None or limited use without consequences	Moderate use with moderate consequences for instance sporadically missing job or social appointments because of substance abuse	Serious abuse with serious consequences, for instance not being able to maintain a paid job, not being able to go to school, physical illness or financial problems because of substance abuse
Aggression Lifetime Last month	None	Verbal aggression or minimal physical aggression without physical harm towards self or others for instance damaging goods or personal belongings	Serious threats and/or physical aggression with physical harm towards self or others
Suicidality Lifetime Last month	No suicidal thoughts ever (thoughts with intent to end life)	Suicidal thoughts present, without suicide plan, no history of attempted suicide or suicidal actions	Suicide plan, history of attempted suicide, suicidal actions
Compliance Lifetime Last month	None or minimal problems	Moderate compliance, for instance infrequent irregular use of medication	Serious problems, for instance severe avoidance of mental health care
Adverse life events	None or minimal adverse life events	Adverse life events without serious physical or emotional threats, violence and/or aggression	Traumatic life events with serious physical, emotional threats, violence and/or aggression
Involuntary treatment	None	One episode of short term involuntary treatment	More than 1 episode of short term involuntary treatment or 1 episode of long term involuntary treatment
Illegal activities	None or minimal, for instance traffic fines	Moderate illegal activities, no imprisonment, no violence for instance stealing a bike	Serious crimes with imprisonment and/or violence
Illness insight Lifetime Last month	Good insight	Moderate insight, for instance recognizing Symptoms, but not psychiatric diagnosis	Minimal to no insight

Study 2 (*n* = 20)

Following findings of insufficient pre‐training IRR in study 1, an instruction manual was developed in which specific criteria for each profile characteristic severity score were described with practical examples and instructions. Two researchers (A.T. and T.B.) thoroughly discussed the instruction manual and independently determined profile characteristics scores based on the formal report.

Study 3 (*n* = 20)

Based on the findings of insufficient IRR of some profile characteristics after introducing an instruction manual and training we adjusted the instruction manual by extending criteria of severity scores. Furthermore, the designated sections in the formal report from which information should be retrieved to determine profile characteristics scores, were described. Profile characteristic specific terms were introduced to explore the report with the search‐function, for example “*school*,” “*work”* or “*physical ill*.” With these adjustments we aimed to ensure that both raters retrieve the same information from the report and base their profile characteristic scores on the same data. Subsequently, two researchers (A.T. and T.B.) independently assessed profile characteristic scores based on a new set of formal reports.

### 2.6 Ethic approval

Data used in the current study were anonymized by an administrative employee who was involved in treatment planning. Both researchers who assessed IRR based on formal reports had no access to non‐anonymized data. Furthermore, the Dutch Central Medical Ethical Committee has ruled that Dutch Law regarding research with humans does not apply to the collection of anonymized information and, consequently, analyzing anonymized data for the present study does not require additional informed consent from participants.

### 2.7 Statistical analysis

To determine the IRR between the two independent researchers we chose the Intraclass Correlation Coefficient (ICC, 2‐way, mixed‐effects model with absolute agreement) with 95% confidence interval. The ICC was chosen as IRR coefficient as our primary endpoint was an ordinal variable. Further, recent literature proposed more strict cut‐off values for interpreting ICC values. Accordingly, we used the following ICC cut‐off values: ICC <0.5 reflects poor agreement, ICC 0.5–0.75 reflects moderate agreement and ICC >0.75 reflects high agreement (Koo & Li, 2016). Analysis of variance or Chi‐Square tests were used to test for differences in gender, age, GAF, use of antipsychotic medication (yes or no) and diagnosis between studies.

## 3 RESULTS

### 3.1 Profile characteristics

A detailed description of the profile characteristics and specifications of severity scores are shown in Table 2. The following profile characteristics were selected: substance use, aggression, suicidality, compliance, insight (lifetime prevalence and prevalence during last month was assessed), work or other activities, support of relatives, physical health, adverse life events, involuntary treatment and illegal activities (prevalence during last month was assessed). Profile characteristics were rated on a 3‐point severity scale: (1) light problems or no problem, (2) moderate problems and (3) severe problems. A score of 0 was assigned in case of missing information.

### 3.2 Inter‐rater reliability

In Table 3 IRR scores of profile characteristics in all studies are shown. In study 1, pre‐training reliability estimates of profile characteristics Involuntary care and Illegal activities were high (ICC: 0.912 and 0.857). IRR scores of the majority of profile characteristics was moderate (ICC: 0.5–0.75), while IRR score of profile characteristics support of relatives and insight recent was low (ICC score: 0.410 and 0.397).

**TABLE 3:** IRR of profile characteristics

Profile characteristic	Study 1 (*n* = 99)	Study 2 (*n* = 20)	Study 3 (*n* = 20)
Involuntary care	**0.912 (0.872–0.940)**	0.653 (0.272–0.855)	**0.968 (0.923–0.987**)
Illegal activities	**0.857 (0.794–0.902**)	**1.000**	**1.000**
Suicidality lifetime	0.743 (0.640–0.820)	0.550 (−0.129–0.868)	**0.916 (0.746–0.975**)
Substance use lifetime	0.696 (0.578–0.786)	**0.897 (0.740–0.961**)	0.555 (0.017–0.820)
Aggression lifetime	0.644 (0.456–0.766)	0.400 (−0.159–0.809)	−0.103 (−0.429–0.323)
Daily activities	0.622 (0.484–0.729)	**0.762 (0.494–0.899**)	0.317 (−0.102–0.661)
Life events	0.579 (0.432–0.696)	**0.777 (0.456–0.919**)	0.231 (−0.142–0.610)
Compliance recent	0.575 (0.423–0.694)	0.391 (−0.025–0.704)	**0.758 (0.480–0.897**)
Physical health	0.548 (0.394–0.672)	0.571 (0.152–0.815)	**0.778 (0.517–0.906**)
Support of relatives	0.410 (0.232–0.562)	0.554 (0.143–0.798)	0.066 (−0.322–0.463)
Insight recent	0.397 (0.217–0.551)	0.424 (0.015–0.722)	0.596 (0.125–0.831)
Aggression recent	‐	0.706 (0.216–0.911)	0.655 (0.317–0.846)
Suicidality recent	‐	0.596 (0.233–0.816)	**0.764 (0.482–0.904**)
Substance use recent	‐	0.678 (0.349–0.859)	0.422 (−0.028–0.725)

Abbreviation: IRR, inter‐rater reliability. In bold ICC scores > 0.75.

In study 2, IRR scores of profile characteristics life events, daily activities, substance use lifetime and illegal activities was high (ICC: 0.762–1.000), whereas most profile characteristics scored moderate reliability (ICC: 0.5–0.75). IRR scores of profile characteristics insight recent, aggression lifetime and compliance recent was low (ICC 0.391–0.424).

In study 3, IRR scores of the profile characteristics Physical Health, Suicidality lifetime, Suicidality recent, Compliance recent, Involuntary care and Illegal activities were high (ICC: 0.758–1.000). Reliability estimates of profile characteristics Substance use lifetime, Insight recent and Aggression recent was moderate (ICC: 0.555–0.655), while IRR scores of remaining four profile characteristics were low.

## 4 DISCUSSION

In the present study we created comprehensive set of prognostic factors that were considered clinically relevant and feasible in regular clinical practice. We investigated the IRR of these profile characteristics in patients with recent onset psychotic disorders. In addition, we explored whether training procedures and scoring instructions improved reliability estimates of profile characteristics.

The selected profile characteristics represent important aspects of symptomatology, comorbidity and psycho‐social functioning relevant for prognosis and treatment in psychosis and related disorders. Our findings showed that the majority of IRR scores of profile characteristics were insufficient, and that training procedures provided some improvement in IRR of some profile characteristics. However, after training, some profile characteristics actually showed reduced IRR.

We found no comparable studies concerning the IRR of profile characteristics, therefore we are not able to compare our results with previous studies. On the other hand, psychiatric classifications such as schizoaffective disorder or attention deficit disorder also show highly variable reliability estimates (Grabemann et al., 2017; Santelmann, Franklin, Busshoff, & Baethge, 2016). Even beyond the scope of psychiatry, the diagnosis of allergic laryngitis or classification systems for scoring tibia plateau fractures demonstrated insufficient or highly diverse IRR scores (Millar, Arnold, Thewlis, Fraysse, & Solomon, 2018; Stachler & Dworkin‐Valenti, 2017). We therefore conclude that IRR of profile characteristics, psychiatric classifications and at least certain diagnoses in general medicine are highly variable.

When examining our results in more detail, pre‐training IRR of profile characteristics in study 1 was generally inadequate, except for the profile characteristics Involuntary Care and Illegal Activities. These results are in line with various research concerning insufficient pre‐training IRR of observational instruments (Berendsen et al., 2019; Muller & Dragicevic, 2003; Muller et al., 1998). Utilizing profile characteristics or other psychiatric instruments without instruction and training for raters should therefore be avoided.

Following implementation of the instruction manual four profile characteristics reached sufficient reliability, while IRR scores of the majority of profile characteristics remained poor. After the manual was complemented by optimizing scoring methods, and adding more detailed specifications and instructions for the raters, several profile characteristics achieved sufficient IRR scores, whereas the observed IRR score of eight profile characteristics remained insufficient.

Previous studies concerning training procedures followed by reliability assessment generally show improvement of IRR. For instance: a commonly applied instrument such as the Positive and Negative Syndrome Scale demonstrated a substantial increase in IRR after training (Kobak et al., 2004; Muller et al., 1998). On the other hand, there is conflicting evidence whether intensive training procedures for the Hamilton Depression Rating Scale (HDRS) actually lead to improvement of the IRR (Demitrack, Faries, Herrera, DeBrota, & Potter, 1998; Muller & Dragicevic, 2003). Various reasons have been stated for the non‐improving reliability estimates of the HDRS after training, for instance rater selection or shortcomings in item‐content.

Despite great effort we were not able to achieve sufficient reliability scores for all profile characteristics, we hypothesize that this could be due to two main reasons. To start, although we aimed to set clear specifications of severity scores in the instruction manual, some profile characteristics might have been insufficiently defined to reach agreement between professionals. For instance, the description of severity scores of the profile characteristic support of relatives may have introduced too much room for interpretation. To illustrate: differentiating between moderate and severe scores, raters had to distinguish infrequent support of relatives (moderate) or serious problems with relatives (severe). The same difficulties in setting distinct boundaries between severity scores were found for the profile characteristics Aggression, Substance abuse recent or Insight recent. Identical concerns were found in achieving sufficient IRR for the HDRS, the authors suggested that limitations in definition of items of the HDRS may have resulted in poor reliability estimates (Demitrack et al., 1998).

Another possible cause of the observed low IRR might be that in the diagnostic report information was missing too frequently to adequately determine profile characteristic scores. Raters might have been obliged to select options based on lacking information, this could have led to an increased information variance which is a major source of low reliability. Nevertheless, our finding of large disagreement among raters when obtaining information from medical records is disturbing, considering the substantial number of studies that are based on collecting information from medical records.

An important question that remains is whether profiling is the best approach to address heterogeneity in psychiatric illness. Profile characteristics are easily applicable for clinicians and not labor intensive. However, it approved difficult to achieve overall reliability in the assessment of the profile characteristics. From that perspective, introduction into clinical practice or scientific research is not warranted at the current time, since insufficient reliability affects study power, placebo response and possible clinical decision making (Berendsen et al., 2019; Kobak et al., 2010; Perkins et al., 2000). Consequently, effort should be focused on improving reliability of the assessment of these prognostic factors.

Our study should be viewed in light of several limitations. First, our sample size in study 2 and 3 is underpowered to permit strong conclusions concerning improvement or deterioration of reliability scores. Therefore, we conducted no formal statistical comparisons between ICC scores of the different studies. We merely tested improvement of ICC scores in the established categories for interpretation of IRR scores as low, moderate to high (Koo & Li, 2016). Second, we only tested IRR. Evaluating other forms of reliability, such as internal consistency or test‐retest agreement is necessary to complement knowledge concerning the psychometric properties of profile characteristics. Third, we did not conduct a systematic search, therefore it is possible that not all studies with prognostic factors for patients with psychotic disorders and related illness were included.

We consider it a strength of our study that we created a comprehensive set of clinically relevant profile characteristics. Another advantage of our study are the training procedures for raters with repeated reliability assessment.

We found that IRR scores of clinical profile characteristics were highly variable and mostly poor, even after training procedures. Therefore, we strongly recommend to further develop definitions and trainings procedures of profile characteristics and evaluate IRR and other psychometric properties before clinical profiling can be introduced in clinical practice. Furthermore, we recommend that future research may explore the relationship between profile characteristics and underlying psychological mechanisms, for instance how the profile characteristic support system may be related to attachment disruptions (Fraley, 2019). Understanding these fundamental mechanisms may support the search for novel targets of therapeutic intervention.

## CONFLICT OF INTERESTS

All authors declare not to have any conflicts of interest that might be interpreted as influencing the content of the manuscript.

## AUTHOR CONTRIBUTIONS

All authors contributed to the study design and proposal, literature search, analysis and interpretation. Authors Amy Tedja and Thijs J. Burger performed the data‐collection, author Steven Berendsen drafted the manuscript and all other authors provided critical revisions. Authors Mirjam J. van Tricht, Mariken B. de Koning and Lieuwe de Haan supervised statistical analysis, study design and writing of the manuscript. All authors contributed to and have approved the final manuscript.
